# Seasonality and daily flight activity of stable flies (Diptera: Muscidae) on dairy farms in Saraburi Province, Thailand

**DOI:** 10.1051/parasite/2013016

**Published:** 2013-05-15

**Authors:** Jumnongjit Phasuk, Atchariya Prabaripai, Theeraphap Chareonviriyaphap

**Affiliations:** 1 Department of Parasitology, Faculty of Veterinary Medicine, Kasetsart University Bangkok 10900 Thailand; 2 Department of Mathematics, Statistics and Computer, Faculty of Liberal Arts and Science, Kasetsart University Kamphaengsean Nakhon Pathom 73140 Thailand; 3 Department of Entomology, Faculty of Agriculture, Kasetsart University Bangkok 10900 Thailand

**Keywords:** *Stomoxys*, daily flight activity, dairy cattle, seasonal abundance, stable flies, Thailand

## Abstract

Knowledge of seasonal abundance and flight activity patterns are required to design effective management programs for insect pests of humans and livestock. In this study, the seasonality and daily flight activity of *Stomoxys* species were observed on two dairy farms in Saraburi Province, Thailand. Data were assessed throughout 1 year using Vavoua traps from September 2010 to August 2011. A total of 2,520 individuals belonging to four species were collected. Most *Stomoxys* species peaked in September (rainy season) and gradually decreased in number toward February (dry season); a second peak occurred between March and April (hot season). *Stomoxys calcitrans* was caught throughout the year and was the most abundant species in this study. The total number of males and females of *S. calcitrans* differed significantly among seasons and time intervals. The weather parameters of relative humidity and light intensity were significantly correlated with *S. calcitrans* abundance.

## Abstract (fr)

La connaissance de l’abondance saisonnière et des patrons d’activité en vol est nécessaire pour établir des programmes efficaces de contrôle des insectes nuisibles à l’homme et au bétail. Dans cette étude, les variations saisonnières et l’activité en vol quotidienne des espèces de *Stomoxys* ont été observées dans deux fermes laitières de la province de Saraburi, Thaïlande. Les données ont été obtenues pendant un an en utilisant des pièges Vavoua de septembre 2010 à août 2011. Un total de 2,520 individus appartenant à quatre espèces a été récolté. La plupart des espèces de *Stomoxys* avaient un pic d’abondance en septembre (saison humide) et leur nombre décroissait régulièrement jusqu’en février (saison sèche) ; un deuxième pic apparaissait entre mars et avril (saison chaude). *Stomoxys calcitrans* a été capturé toute l’année et était l’espèce la plus abondante dans cette étude. Le nombre total de mâles et de femelles de *S. calcitrans* différait significativement selon les saisons et les intervalles de temps. Les paramètres climatiques d’humidité relative et d’intensité lumineuse étaient corrélés de manière significative avec l’abondance de *S. calcitrans*.

## Introduction

Stable flies belong to the subfamily Stomoxyinae in the family Muscidae (Diptera). Among 18 *Stomoxys* species described, six species are recorded from Thailand, of which one is cosmopolitan, *S. calcitrans* (Linnaeus, 1758) [9, 22, 24]. Stable flies resemble house flies but can be easily distinguished by their piercing-sucking mouthparts which are conspicuous, long and project straightforward from under the head. They are important and widely distributed insect pests of livestock, wildlife and sometimes humans. Adult stable flies of both sexes are blood-sucking flies and cause painful bites and significant blood loss in some animals. High populations of biting activity can reduce animal productivity and disturb feeding resulting in reduced weight gain and milk production [2–5, 23]. Moreover, they may act as both biological and mechanical vectors for pathogens such as trypanosomes [19]. Stable flies may also act as an intermediate host of the nematode *Habronema* [21]. In Thailand, little is known about the presence of different stomoxyine fly species, their distribution and their biology. However, Masmeatathip *et al*. [11] and Muenworn *et al*. [13] described the seasonal abundance and daily activity of *Stomoxys* species in Thailand. Muenworn *et al*. [12, 13] conducted stable fly surveys and reported their distribution in Thailand. A better understanding of the seasonal and daily activity of the flies will facilitate and make fly control programs more effective. This study had the objective to evaluate the seasonal abundance and daily activity of *Stomoxys* species.

## Materials and methods

### Study sites

**Figure 1.**
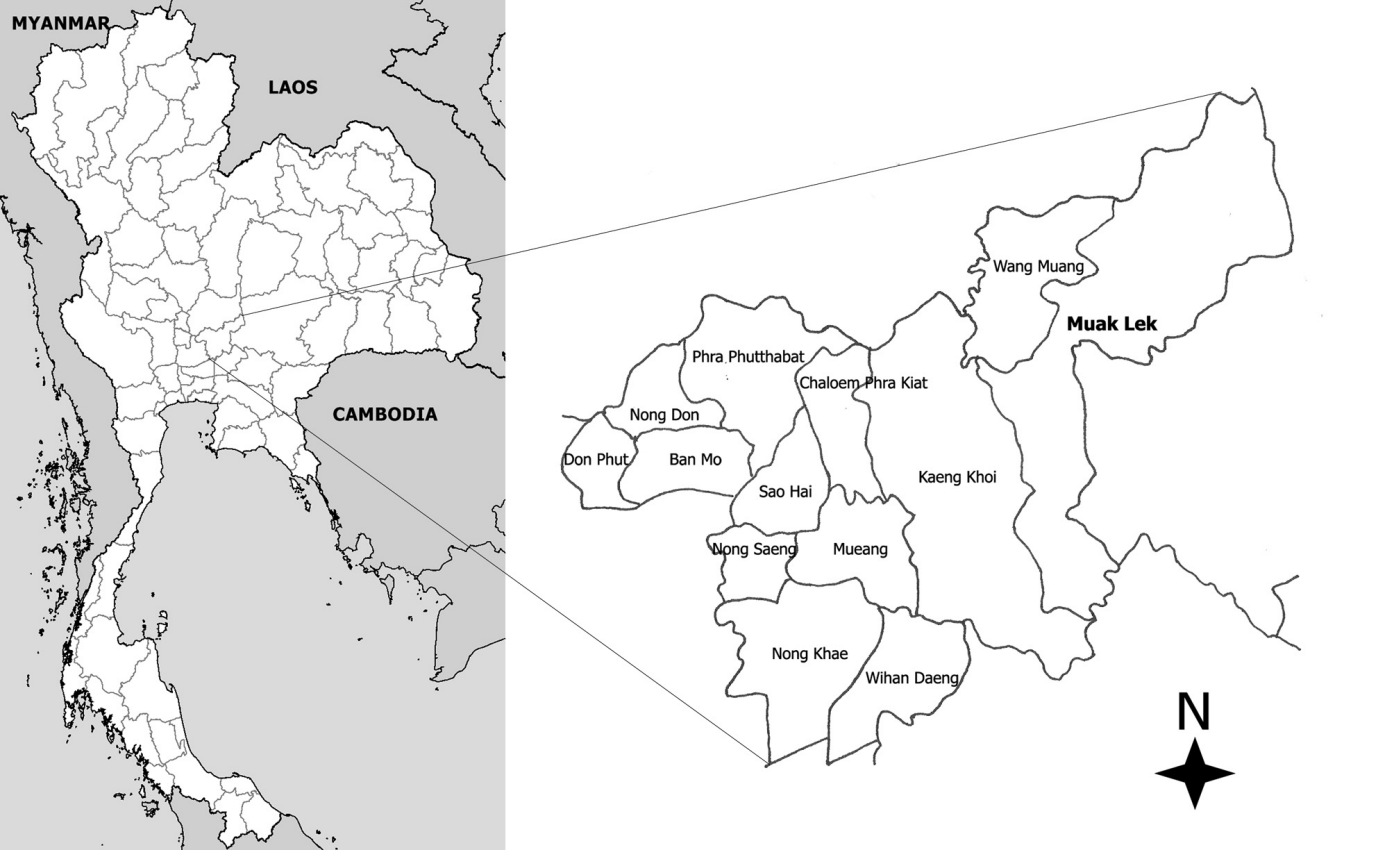
Location of Muak Lek district (study area) in Saraburi Province, Thailand.

This study was conducted on two dairy farms in Muak Lek district, Saraburi Province (Figure 1). Saraburi is located in the central highlands of Thailand, an area of relatively high plains and plateaus about 140 km Northeast of Bangkok. The study sites are 355 and 460 m above sea level (a.s.l.). Thailand has three seasons: hot (March to May), rainy (June to October) and dry (November to February). At the time of the study, farm 1 (14° 48′ N, 101° 17′ E; 460 m a.s.l) had 10 cows and farm 2 (14° 47′ N, 101° 15′ E; 355 m a.s.l) had 57 cows in milk production.

### Specimen collection

At each site, four Vavoua traps [8] were randomly installed around sample sites at least 5 m apart from each other. The traps were placed at 5–10 cm above the ground. Stable flies were observed monthly during a one-year study (September 2010 to August 2011). Flies were captured every hour between 0600 and 1800 h. After trapping, flies in the Vavoua traps were killed using ethanol spray and preserved in 80% ethanol. Air temperature, relative humidity and light intensity were also recorded every hour by using a digital thermometer and a digital light meter at each farm. The specimens were brought back to the Department of Parasitology, Faculty of Veterinary Medicine, Kasetsart University, Bangkok, Thailand, for identification using the keys of Zumpt [24] and Tumrasvin & Shinonaga [22].

### Data analysis

The total numbers of each sex of stable flies collected from traps at each collection site were analyzed using a one-way analysis of variance (ANOVA) and the time intervals were considered as treatment effects. Variations in the numbers of each sex between seasons were compared using the least significant difference (LSD) test. Stepwise multiple regression analysis was used to determine the relationship between the abundance of stable fly species and weather parameters. The data were analyzed using SPSS (Version 17, SPSS Inc., Chicago, IL, USA). All statistical significance levels were set at *p* < 0.05.

## Results

In total, 2,520 individuals of *Stomoxys* species were collected during the one-year study, of which 1,622 were trapped on farm 1 and 898 on farm 2 (Table 1). The following four species were captured: *S. bengalensis* Picard, 1908 [15], *S. calcitrans* (Linnaeus, 1758) [9], *S. indicus* Picard, 1908 [15] and *S. sitiens* Rondani, 1873 [17].

**Table 1.** Total numbers of *Stomoxys* spp. collected and their relative abundance (RA) on two dairy farms, Muak Lek district, Saraburi Province from September 2010 to August 2011.

Species	Sex	Farm 1	RA (%)	Farm 2	RA (%)
*S. bengalensis* Picard, 1908	Male	0	0	1	0.1
	Female	0	0	0	0
*S. calcitrans* (Linnaeus, 1758)	Male	1,206	74.4	535	59.6
	Female	355	21.9	268	29.8
*S. indicus* Picard, 1908	Male	19	1.2	12	1.3
	Female	34	2.1	25	2.8
*S. sitiens* Rondani, 1873	Male	5	0.3	24	2.7
	Female	3	0.2	33	3.7
Species/total number		3/1,622		4/898	

**Figure 2.**
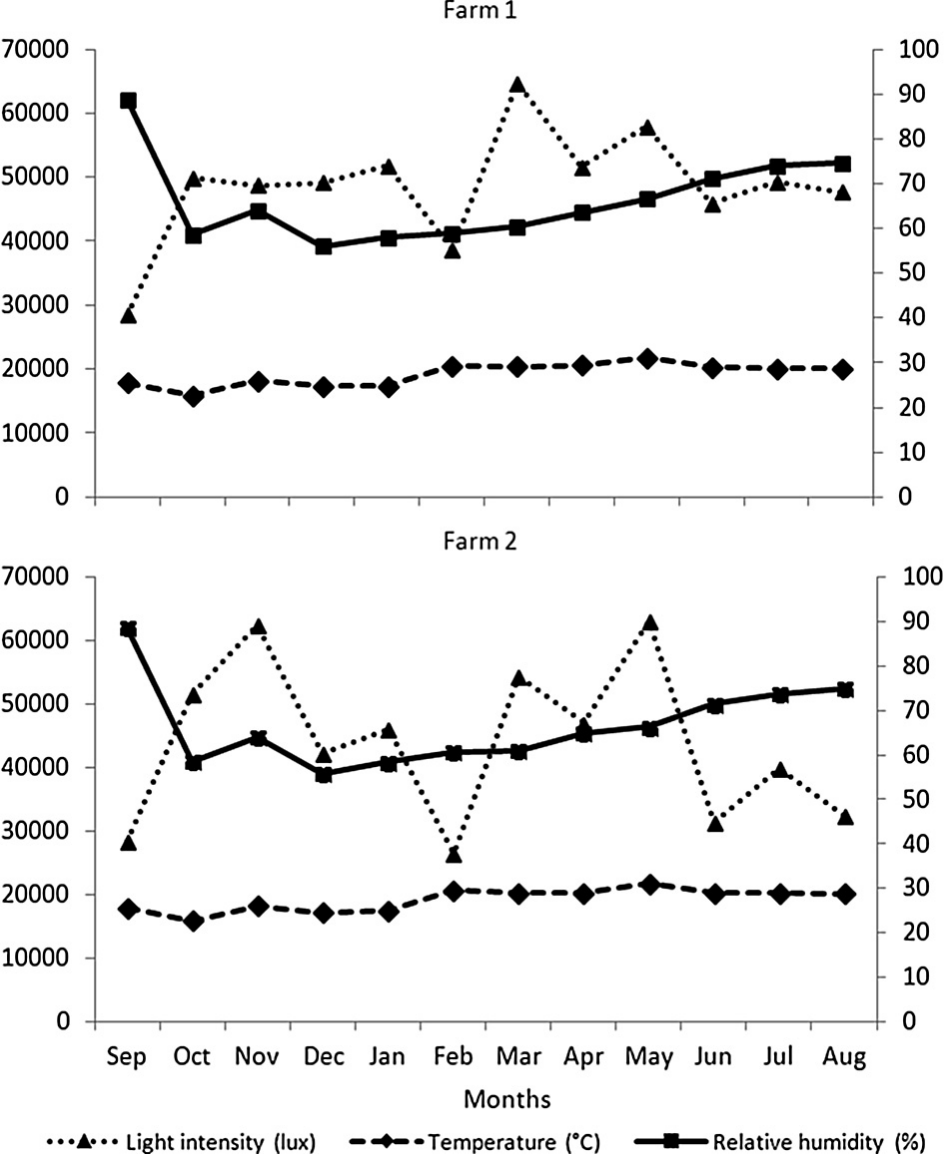
Mean monthly temperature and relative humidity (right axis scale) and light intensity (left axis scale) on the experimental sites (two dairy farms) from September 2010 to August 2011.

**Figure 3.**
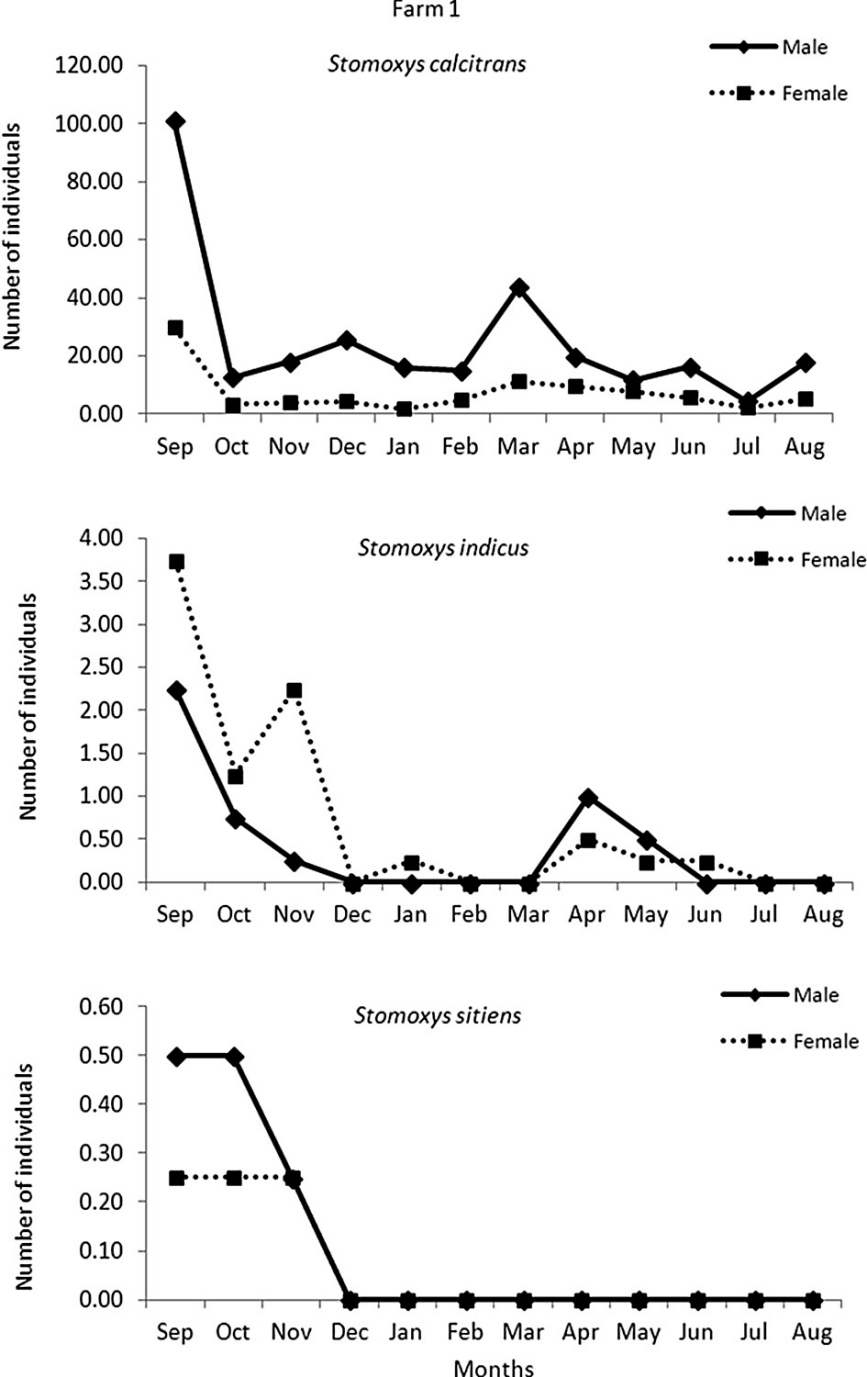
Mean number of *Stomoxys* spp. captured in four traps on dairy farm 1 from September 2010 to August 2011.

**Figure 4.**
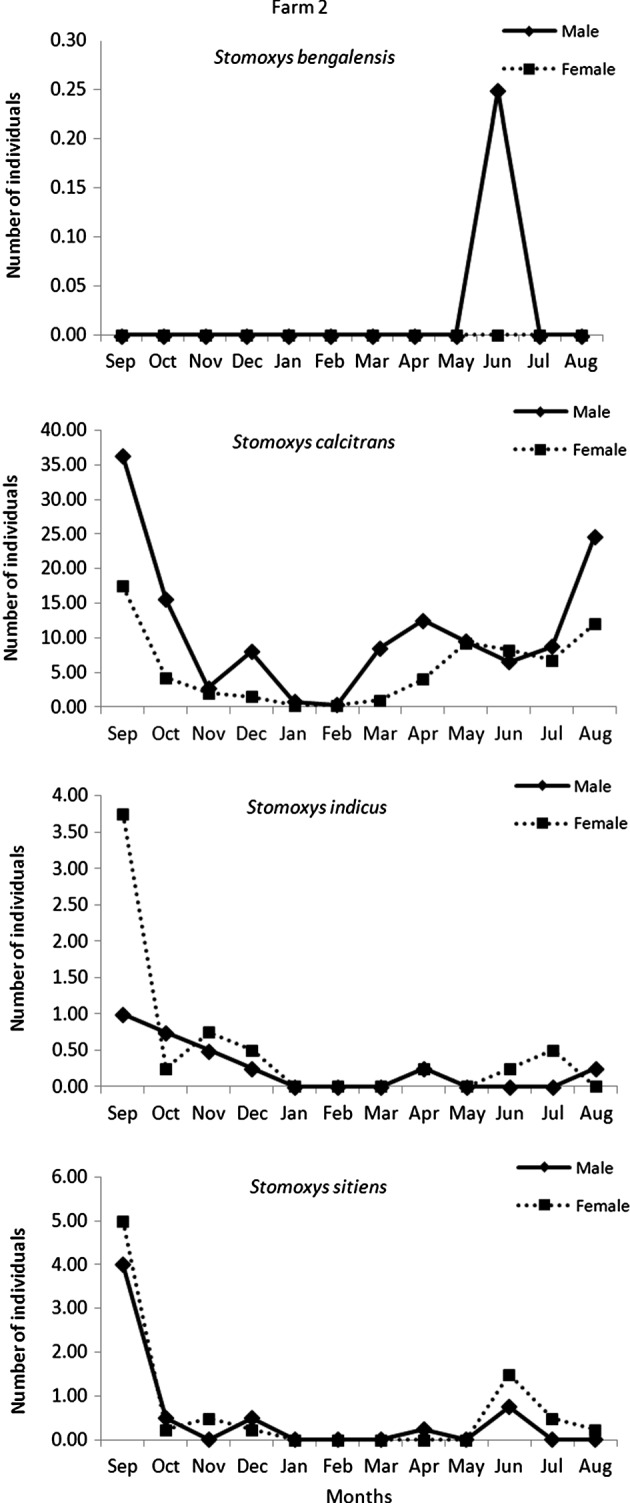
Mean number of *Stomoxys* spp. captured in four traps on dairy farm 2 from September 2010 to August 2011.

The highest temperature was recorded in May and the lowest temperature was in October. The average relative humidity was lower in December to January during the dry season and the highest relative humidity was in September. The average light intensity was high in March to May during the hot season (Figure 2). Seasonal variations in the abundance of *Stomoxys* species are presented at the species and site levels in Figures 3 and 4. The highest numbers of each species were recorded in September and gradually decreased in number toward February. The second peak of most species occurred between March and April. The most abundant species was *S. calcitrans* (89–96%) which was found on both farms. *S. calcitrans* was present throughout the year. Collections of other species were too low to be meaningful for the analysis (Table 1 and Figure 7).

**Figure 5.**
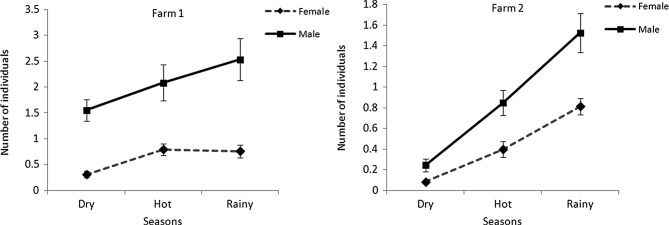
Mean numbers ± standard error bars of *Stomoxys calcitrans* males and females collected during three seasons on two dairy farms.

**Figure 6.**
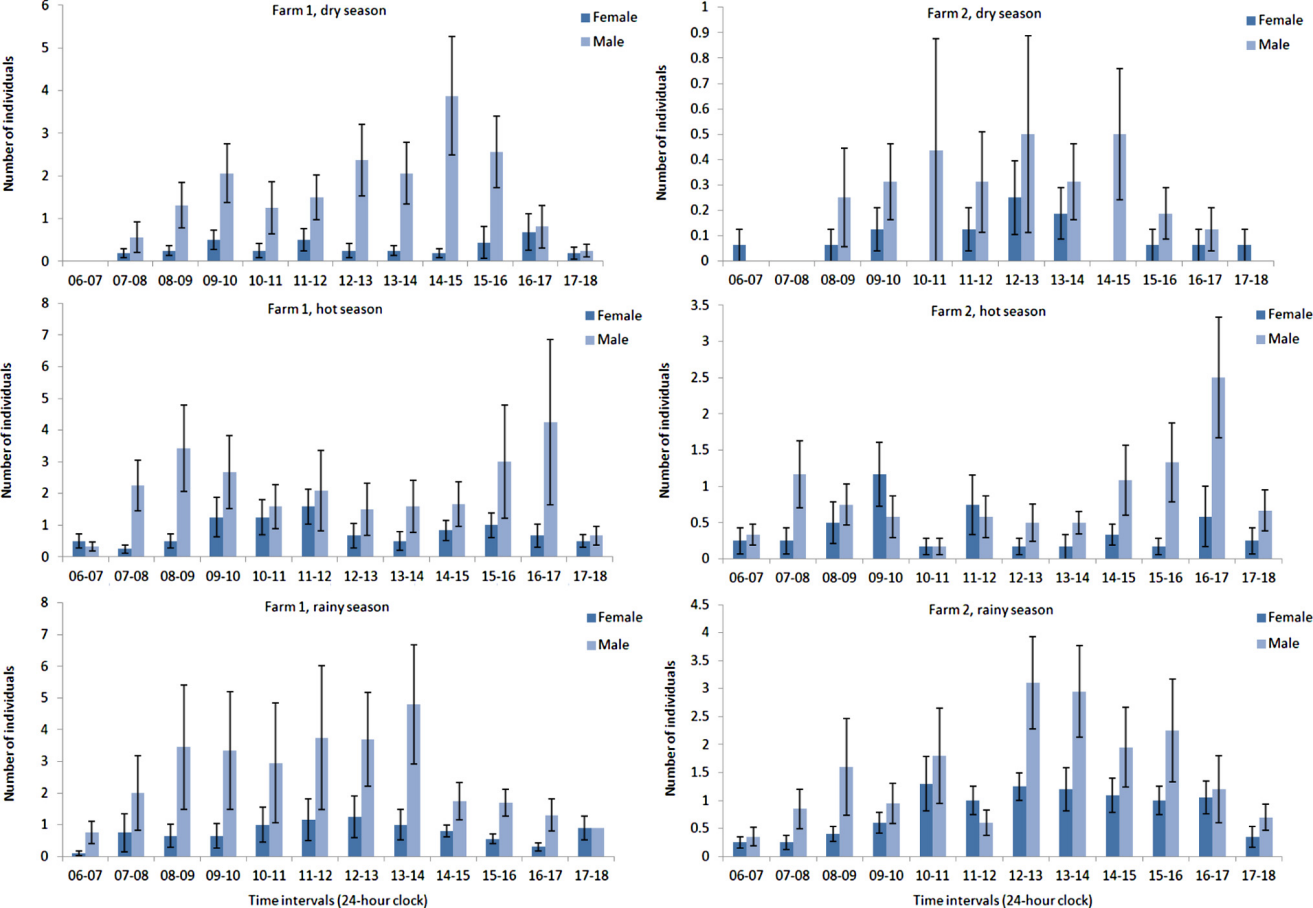
Mean numbers ± standard error bars of *Stomoxys calcitrans* males and females collected during one-hour intervals in three seasons on two dairy farms.

**Figure 7.**
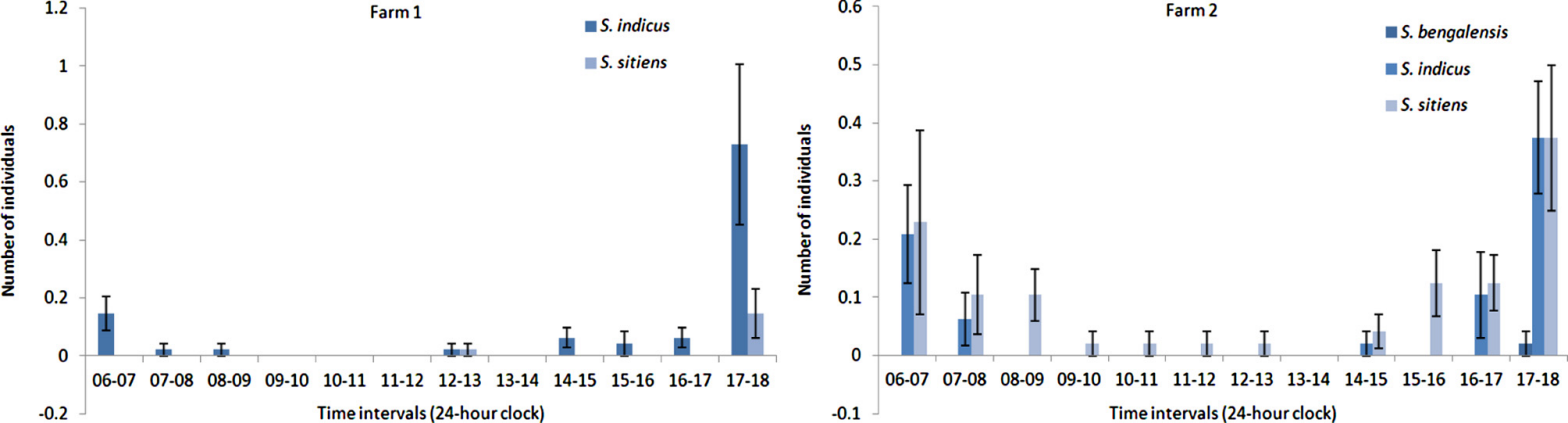
Mean numbers ± standard error bars of *Stomoxys* spp. males and females collected during one-hour intervals on two dairy farms from September 2010 to August 2011.

On both farms, the numbers of males of *S. calcitrans* were significantly (*p* < 0.05) greater than the females in each season (Figure 5). On farm 1, seasonal variations in the numbers of females were significant (*p* < 0.05), except between the hot and rainy seasons, whereas for males, seasonal variations were significant (*p* < 0.05), except between the hot and dry seasons and the hot and rainy seasons. On farm 2, seasonal variations in the numbers of both sexes were significant (*p* < 0.05). The abundance of *S. calcitrans* peaked in the rainy season and declined in the dry season.

On farm 1, the total number of females of *S. calcitrans* collected did not differ significantly among time intervals in the dry, hot and rainy seasons. The numbers of males collected showed a significant (*p* = 0.005) difference among time intervals in the dry season, but there was no significant difference among time intervals in the hot and rainy seasons (Figure 6). On farm 2, the total number of females collected showed a significant (*p* = 0.009) difference among time intervals in the rainy season, but there was no significant difference among time intervals in the dry and hot seasons. Males showed significant differences in time intervals between the hot and rainy seasons (*p* = 0.007, *p* = 0.028, respectively), but not in the dry season (Figure 6).

During the whole collection period, *S. calcitrans* abundance was not related to air temperature but showed a positive correlation with relative humidity and light intensity (Table 2).

**Table 2.** Stepwise multiple regression analysis with correlation between total number of *Stomoxys calcitrans* and weather parameters in Muak Lek district, Saraburi Province from September 2010 to August 2011.

Effect	*b*	*SE* of *b*	Beta	*t*	*p*	
Intercept	−17.266	4.57		−3.778	0.000	
Relative humidity (%)	0.337	0.062	0.336	5.481	0.000	
Light intensity (lux)	0.00006	0.000	0.208	3.39	0.001	
	*R*^2^	*SS*	*df*	*MS*	*F*	*p*
	0.1	3,945.72	2	1,972.86	15.89	0.000

## Discussion

The present study extends our understanding of the seasonal abundance and daily activity of stable flies in Thailand. In Saraburi Province, there were four species in the genera *Stomoxys* collected from both farms and *S. calcitrans* was active throughout the year. Muenworn *et al*. [13] found *S. calcitrans* to be the most abundant species followed by *S. indicus. Stomoxys* populations peaked in September, corresponding to the rainy season. This finding agreed with earlier work by Masmeatathip *et al*. [11] and Muenworn *et al*. [13]. This may have been due to an increase in the rainfall causing a widespread increase in suitable breeding sites which is a critical factor for eggs to hatch and the larvae to survive and successfully develop to pupae and adults. Earlier work shows that immature stages of *Stomoxys* (eggs and larvae) are highly sensitive to the following environmental conditions to survive and successfully develop to pupae and adults: temperature, humidity and rainfall [6, 18].

The numbers of male *S. calcitrans* were significantly higher than females, in agreement with the reports of Masmeatathip *et al*. [11] and Muenworn *et al*. [13]. More female *S. indicus* were collected than males on both farms. Similar findings were reported by Masmeatathip *et al*. [11].The sex ratios of *S. sitiens* varied between farms. Only one male of *S. bengalensis* was seen and that was on farm 2. The variation in the abundance of *Stomoxys* species that were observed may have depended on the temperature, precipitation, types of trap, trap locations and trap height [1, 7, 10, 16]. Further studies are needed to evaluate whether these differences are associated with the location or other factors that attract *Stomoxys* species.

The season seemed to play a major role in the daily activity patterns of *S. calcitrans*. In the dry and rainy seasons, the number of *S. calcitrans* increased throughout the day until 1500 h and gradually decreased in number toward 1800 h. In the hot season, this species was present throughout the day and peaked in abundance in the evening and a secondary peak occurred in the late morning although the numbers of females were not statistically significant among the time intervals. In previous studies, *S. calcitrans* was reported to have a bimodal pattern of feeding with a major peak during 0800–1000 h and a minor peak occurred during the afternoon [11]. Muenworn *et al*. [13] showed a bimodal activity of male *S. calcitrans* with peaks at 0800–1000 and 1400–1600 h while female activity increased throughout the day until 1600 h, when the activity of both males and females declined steadily until it was dark. Masmeatathip *et al*. [11] reported bimodal activity of *S. indicus* and *S. sitiens* with population peaks in the early morning and in the late afternoon. This finding was similar to that observed in this study. Stable flies require blood for successful mating and ovarian development, but also require nectar as a supplemental energy source for flight activity and successful blood-feeding [20]. Müller *et al*. [14] conducted studies on the diurnal feeding behavior of three *Stomoxys* species in Mali and found bimodal blood-feeding and unimodal sugar-feeding activity periods. The differences in feeding activity patterns were dependent upon the protein and nectar sources, local conditions, sampling methods and seasons. Analysis of the data showed the abundance of *S. calcitrans* was significantly and positively correlated with relative humidity and light intensity although the relationship was weak (*R* = 0.317). There were probably other factors that affected the flight activity patterns of this species. The current study has provided a step in the evolution of planning and developing control systems for insects.
